# Shared genetic architecture between grip strength and cognitive function: insights from large-scale genome-wide cross-trait analysis

**DOI:** 10.3389/fgene.2025.1538308

**Published:** 2025-08-14

**Authors:** Hong Liu, Gangqiang Wu, Jun Tan, Chunyun Yuan

**Affiliations:** ^1^ Graduate School, Hunan University of Chinese Medicine, Changsha, China; ^2^ Department of Geriatrics, Hunan Provincial Hospital of Integrated Traditional Chinese and Western Medicine (The Affiliated Hospital of Hunan Academy of Traditional Chinese Medicine), Changsha, China

**Keywords:** genetic correlation, shared genetic architecture, grip strength, cognitive function, aging

## Abstract

**Background:**

Physical and cognitive decline are common in older individuals, and traits related to grip strength and cognitive function are used to assess the common genetic structure between the two and to identify common risk loci and genes as well as the genetic mechanisms involved.

**Methods:**

On the basis of large-scale genome-wide association study (GWAS) summary-level datasets, we observed genetic overlaps between grip strength and cognitive function, and cross-trait pleiotropic analysis was performed to detect shared pleiotropic loci and genes. A series of functional annotations and tissue-specific analyses were performed to determine the influence of pleiotropic genes. Heritance enrichment analysis was used to detect crucial immune cells and tissues. Finally, drug targets were explored via the SMR method.

**Results:**

This study highlighted genetic mechanisms shared between five types of cognitive function-related traits and grip strength. This study identified 20 novel SNP loci (P < 5 × 10^−8^/5) and 7 pleiotropic genomic risk loci, of which 1p34.2 and 4q24 have been shown to be associated with trait pairs in previous studies. Furthermore, 7 unique pleiotropic genes, such as BANK1, CADM2, AFF3 and AUTS2, were identified at the gene level. Four drug targets in European populations were identified via PLACO analysis combined with FUMA, MAGMA and SMR results, which were consistent with the pleiotropic genetic results and were novel. Finally, the immune mechanisms of trait pairs were validated via HyPrColoc.

**Conclusion:**

Overall, our results provide new insights into the genetics of cognitive function and grip strength and shed light on the underlying molecular mechanisms that may be involved.

## Background

Physical and cognitive decline is common in the elderly population, and current studies suggest that a decline in physical function may have an impact on cognitive function through mechanisms such as muscle-derived mediators that mediate muscle‒brain crosstalk and shared neural substrates (Arosio et al., 2023; Buchman et al., 2020; Buchman et al., 2019). Grip strength is one of the main indicators for assessing physical function, with the advantages of being reliable, simple, rapid and inexpensive, and some studies have suggested that grip strength can be used as an indicator for monitoring cognitive decline (Peolsson and Oberg, 2001; Bohannon, 2019; hou et al., 2019; strength, 2015; Fritz and Adamo, 2017).

Epidemiological investigations have revealed associations between grip strength and cognitive impairment. For example, a study that included 9,333 adults aged 45+ years from the China Health and Retirement Longitudinal Study (CHARLS) reported that a higher baseline level of grip strength was significantly related to better cognitive function and slowed the rate of its decline (Liu et al., 2019); a study that included 6,806 adults aged 50+ years from the Guangzhou Biobank Cohort Study (GBCS) reported that grip strength was positively associated with delayed word recall test and recall memory performance but not general cognitive function (Jin et al., 2022); a study of 2,623 elderly individuals aged 60+ years reported a negative correlation between grip strength and cognitive impairment via the digit symbol substitution test (DSST) (Huang et al., 2022).

There are few studies that use grip strength as an independent biomarker and discuss it in relation to cognitive function, and most include it in related phenotypes such as sarcopenia and frailty (Sui et al., 2022; Lu et al., 2024; Sharifi et al., 2021). This exemplifies the inadequacy of current research on the associations between grip strength and cognitive impairment, which involves clinical or epidemiological studies, potential mechanisms and drug target exploration.

**FIGURE 1:**
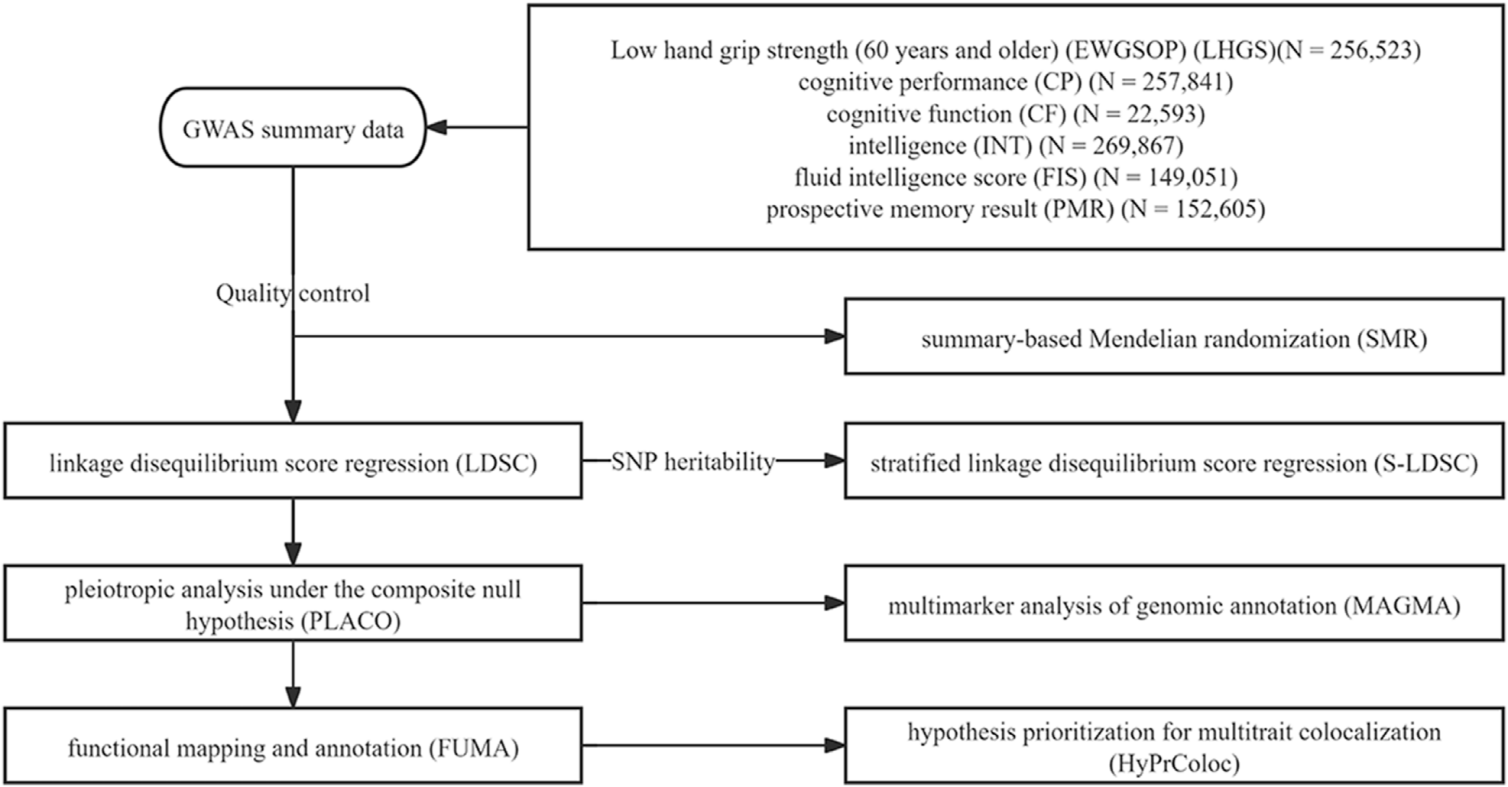
Study workflow. This study aims to investigate the shared genetic structure and potential molecular mechanisms between low hand grip strength (LHGS) and cognitive function traits, including cognitive performance, intelligence, and fluid intelligence scores. Based on large-scale genome-wide association studies (GWAS) data from European populations, the study uses linkage disequilibrium score regression (LDSC) to analyze genetic structure, stratified LDSC (S-LDSC) for tissue enrichment analysis, pleiotropic analysis under the composite null hypothesis (PLACO) to identify pleiotropic SNP, functional mapping and annotation (FUMA) and multimarker analysis of genomic annotation (MAGMA) for gene or tissue-level analysis, summary-based Mendelian randomization (SMR) for potential drug targets, and HyPrColoc to explore immune mechanisms. The ultimate goal is to uncover the shared genetic loci, genes, and potential therapeutic targets between the two.

Therefore, it is necessary to further explore the association between grip strength and cognitive impairment through innovative approaches. Novel methods such as linkage disequilibrium score regression (LDSC) and pleiotropic analysis under the composite null hypothesis (PLACO) could be employed to identify specific genetic variants or loci contributing to genetic correlations. Such investigations are crucial for revealing the underlying mechanisms of genetic correlation, elucidating shared genetic structures across phenotypes, and discovering potential therapeutic or preventive targets (Bulik-Sullivan et al., 2015; Yu et al., 2024; Ray and Chatterjee, 2020). Our research flowchart is shown in Figure 1.

## Methods

### Data sources

The GWAS summary statistics for the five metrics related to the assessment of cognitive impairment were taken from large-scale GWAS or GWAS meta-analyses, all obtained from the IEU Open GWAS project (https://gwas.mrcieu.ac.uk/, accessed 11 August 2024): cognitive performance (CP) (ebi-a-GCST006572) (Lee et al., 2018), cognitive function (CF) (ieu-b-4838), intelligence (INT) (ebi-a-GCST006250) (Savage et al., 2018), fluid intelligence score (FIS) (ukb-b-5238), and prospective memory result (PMR) (ukb-b-4282).

The GWAS summary statistics for low grip strength were derived from a genome-wide meta-analysis of 22 independent cohorts. The study screened 256,523 individuals of European ancestry aged 60 years or older on the basis of the European Working Group on Sarcopenia in Older People (EWGSOP) definition of low grip strength (Jones et al., 2021).

The data on LHGS, CP, and INT were derived from genome-wide meta-analysis involving 22, 71 and 14 GWAS cohorts, respectively. These meta-analyses implemented stringent quality control protocols for all cohorts to ensure the accuracy of genetic variation results. Each GWAS cohort was analyzed using either a linear regression model or a logistic regression model, with adjustments for age, sex, and cohort-specific covariates related to genetic principal components. The Meta analysis was conducted using METAL software, integrating results through fixed effect inverse variance weighting (IVW) or sample size weighting methods. For some polygenic analyses, non-central parameter weighting was used to optimize the combination of effect values. The data sources are detailed in Additional file 1: Supplementary Table S1.

### Quality control

To improve statistical validity and reduce false positives, the study excluded SNPs in the major histocompatibility complex (MHC) (chromosome 6: 25–35 megabases [Mb]) and retained only SNPs with a minor allele frequency (MAF) greater than 0.01.

### Genome-wide genetic correlation

We used LDSC to evaluate the genetic structure shared between LHGS and cognitive impairment (Bulik-Sullivan et al., 2015). The LDSC framework incorporated LD scores computed from common SNP genotypes obtained through European-ancestry samples in the 1,000 Genomes Project (Auton et al., 2015). Within this methodology, jackknife resampling was applied to derive to derive standard error (SE) estimates that are robust to sample overlap between the measured traits. The LDSC intercept values served as quantitative indicators to assess potential population stratification bias; results confirmed some traits did not show signs of significant population stratification meaning that the GWAS signal is likely to represent true polygenic signal for all of the measured phenotypes. Crucially, both GWAS cohorts were derived from European-ancestry populations, maintaining genetic background homogeneity to support valid correlation analysis.

### Tissue-level exploratory analyses

We used a stratified LDSC (S-LDSC) regression approach to investigate the extent to which SNP heritability for LHGS and cognitive impairment were associated in different tissues. We downloaded 54 human tissue datasets from GTEx (GTEx Consortium, 2013) and assessed the significance of SNP heritability enrichment in different tissues.

### SNP-level exploratory analyses

Our analytical framework implemented pleiotropic SNP prioritization separately between LHGS and each cognitive impairment trait through PLACO, retaining variants surpassing genome-wide significance (P < 5 × 10^−8^/5) as considered pleiotropic variants.

Then, we employed the functional mapping and annotation (FUMA) of GWASs for functional characterization and locus definition of risk-associated variants (Watanabe et al., 2017). Subsequently, Bayesian colocalization analysis was applied to statistically resolve shared causal loci between LHGS and cognitive impairment phenotypes (Giambartolomei et al., 2014).

### Gene-level exploratory analyses

For mechanistic exploration of pleiotropic genomic risk loci which identified by PLACO and FUMA, proximal genes were systematically annotated using lead SNPs from respective genomic regions. Complementarily, the multimarker analysis of genomic annotation (MAGMA) framework was implemented for functional ontology characterization of pleiotropic loci through GWAS data interrogation.

Specifically, we performed MAGMA gene analysis to identify pleiotropic genes by properly incorporating LD between markers and to detect multimarker effects (de Leeuw et al., 2015). Subsequent gene set interrogation leveraged the MAGMA framework to functionally annotate lead SNPs, examining 10,678 biological pathways comprising curated gene sets (c2.all) and GO terms (c5.bp, c5.cc, and c5.mf) from the Molecular Signatures Database (MSigDB), were finally tested (de Leeuw et al., 2015; Subramanian et al., 2005). All pathway associations underwent stringent multiple testing correction via the Bonferroni method to ensure statistical robustness.

Tissue-specific enrichment profiling leveraged 54 GTEx v8 tissues (Carithers et al., 2015) to annotate PLACO-derived pleiotropic signals across the genome. Complementary expression profiling quantified log2-transformed mean expression levels of pleiotropic genes across matched GTEx tissues; tissue specificity was systematically interrogated through predefined differential expression signatures (|t-statistic| ≥ threshold for DEG classification).

### Exploration of potential drug targets in european populations

The summary-based Mendelian randomization (SMR) framework (Zhu et al., 2016) integrates summary-level GWAS and expression quantitative trait loci (eQTL) datasets to prioritize genes demonstrating pleiotropic associations between transcriptional activity and complex traits. The analytical framework implements dual SMR-HEIDI testing to interrogate expression-phenotype pleiotropy, simultaneously evaluating whether phenotypic SNP effects are transcriptionally mediated. Furthermore, this approach elucidates gene regulatory mechanisms linking genetic variation to phenotypic outcomes, thereby prioritizing druggable targets for therapeutic exploration.

### Multitrait colocalization analysis

Our investigation employed hypothesis prioritization for multitrait colocalization (HyPrColoc) (Foley et al., 2021) to conduct cross-phenotype colocalization analyses, which included GWAS data of LHGS, immunogenomic dataset of immune cells, and loci identified by PLACO and FUMA, delineating the immune signatures of pleiotropic genomic risk loci.

Our study used the hypothesis priority of multi-feature co-location (HyPrColoc) for cross-phenotype co-location analysis, and to define the immune characteristics of polygenic risk loci.

This framework demonstrates enhanced statistical power for resolving immune-mediated etiological pathways in complex diseases while robustly identifying candidate immune regulatory architectures. Furthermore, it aims to provide new insights into the systemic immune modulation mechanisms underlying LHGS-associated cognitive decline.

The analyzed immunogenomic dataset comprised 731 immune cell traits curated from GWAS catalog entries (GCST0001391–GCST0002121) (Orrù et al., 2020).

## Results

### Genetic correlation between LHGS and cognitive impairment

We first assessed the genetic correlation between LHGS and cognitive impairment via the LDSC method. Specifically, it was identified to be genetically correlated between CP, INT and FIS and LHGS (p < 0.05/5) (Table 1).

**TABLE 1:** Genetic correlation between LHGS and cognitive impairment.

Trait pairs	LDSC	
	r_g_ (SE)	P
LHGS&CP	−0.0869 (0.0275)	0.001575
LHGS&CF	−0.1367 (0.05692)	0.01631
LHGS&INT	−0.07709 (0.02779)	0.005529
LHGS&FIS	−0.08808 (0.02934)	0.002682
LHGS&PMR	0.1049 (0.04248)	0.0135

Trait abbreviations: LDSC, linkage disequilibrium score regression; LHGS, low hand grip strength; CP, cognitive performance; CF, cognitive function; INT, intelligence; FIS, fluid intelligence score; PMR, prospective memory result.

### Tissue enrichment associated with SNP heritability

We further investigated the genetic enrichment of individual traits in specific tissues via the S-LDSC method. We downloaded a dataset of 54 human tissues from GTEx and assessed the significance of SNP heritability enrichment in each tissue by regression coefficient z scores and corresponding p values after adjusting for the baseline model and all gene sets.

**FIGURE 2:**
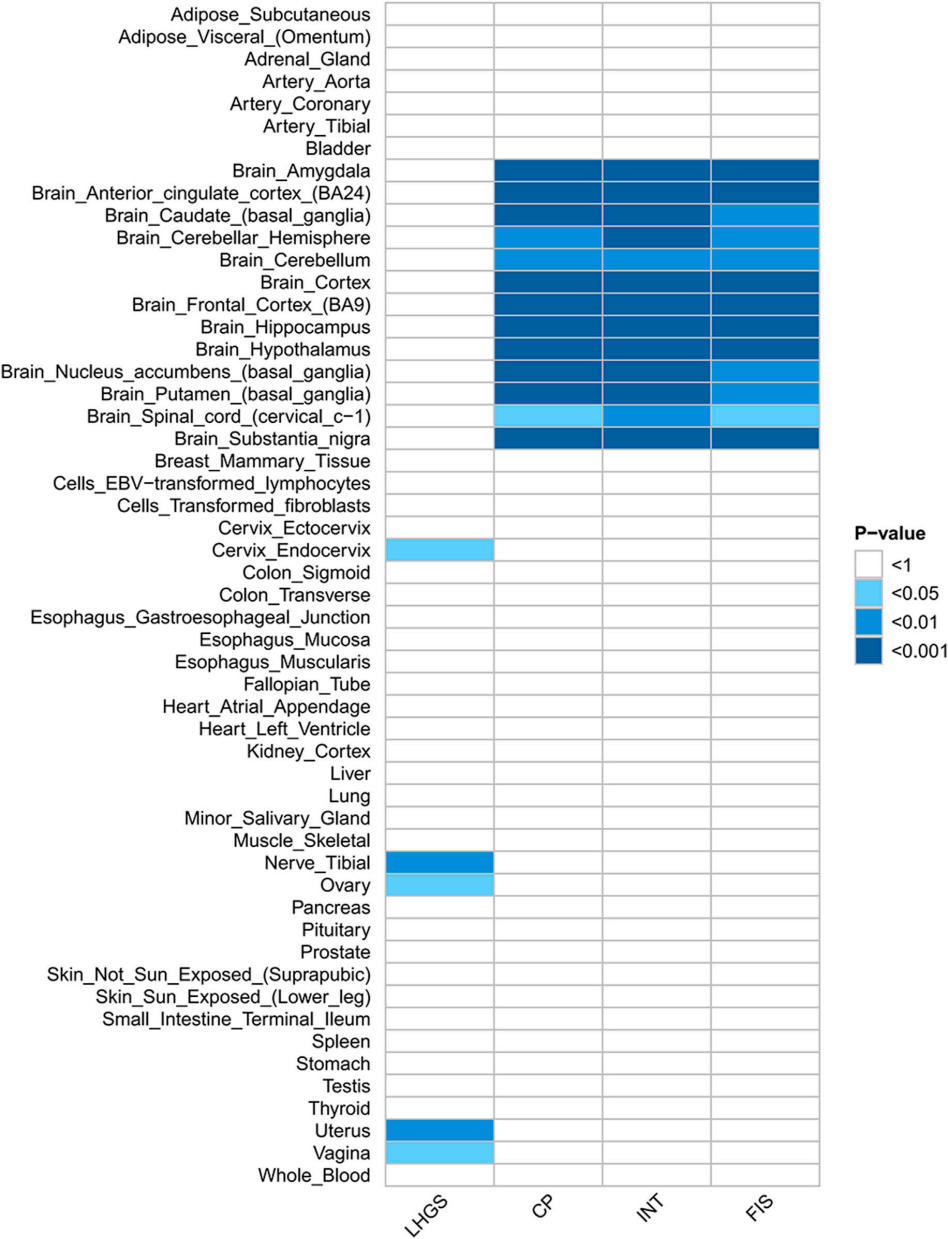
Heatmap of heritability enrichment identified by S-LDSC. The heatmap visually shows the significance of heritability enrichment of LHGS and cognitive function-related traits in different tissues by color depth: the darker the color, the smaller the P value (corresponding to P < 0.05, 0.01, 0.001 successively), and the blank area indicates that no significant heritability enrichment is detected. The core findings of this figure are as follows: the heritability tissue enrichment results of cognitive impairment-related traits and LHGS are inconsistent, suggesting that this result cannot be used as evidence of direct genetic correlation between the two at the tissue level. Trait abbreviations: LHGS–low hand grip strength; CP–cognitive performance; INT–intelligence; FIS–fluid intelligence score.

Further tissue-specific analyses revealed that these SNP loci were enriched in some of the tissues but cognitive impairment-related traits were enriched in brain tissue, and LHGS was mainly related to smooth muscle (Figure 2, Additional file 1: Supplementary Table S2).

### Identification of pleiotropic loci

Given the shared genetic mechanisms between multiple traits identified by the LDSC methods, we used PLACO to identify potential pleiotropic loci associated with diseases. In total, we identified 20 novel SNP loci (P < 5 × 10^−8^/5) (Additional file 1: Supplementary Table S3).

On the basis of the PLACO results, we identified a total of 7 pleiotropic genomic risk loci via the FUMA tool; at the same time, a locus with PP.H4 > 0.7 was identified in LHGS-INT trait pairs by colocalization analyses (Additional file 1: Supplementary Table S4).

**FIGURE 3:**
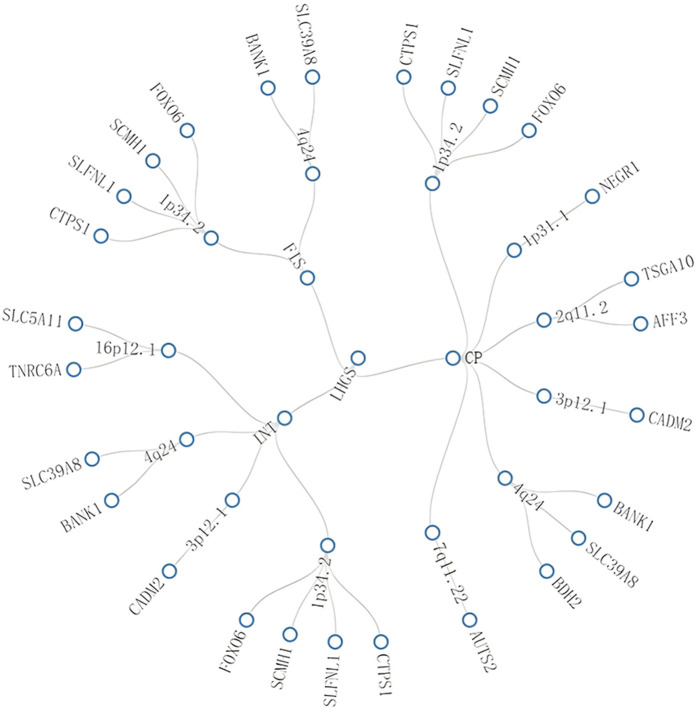
Network graph of multi-effect genomic regions shared between different trait pairs. This figure visualizes associations among traits, genomic regions, and genes through connecting lines, where “multi-effect” refers to a single genetic locus that affects multiple seemingly unrelated traits. The core findings of this figure are as follows: specific regions (e.g., 1p34.2, 4q24) are shared across multiple trait pairs, with genes such as BANK1 implicated in these pleiotropic regions, highlighting shared genetic architecture between LHGS and cognitive function-related traits. Trait abbreviations: LHGS–low hand grip strength; CP–cognitive performance; INT–intelligence; FIS–fluid intelligence score.

Notably, some pleiotropic regions were shared between different trait pairs, e.g., 1p34.2 and 4q24 were identified in three trait pairs (Figure 3, Additional file 1: Supplementary Table S5).

### MAGMA gene set and tissue-specific analysis

Based on the novel SNP loci identified by PLACO, MAGMA gene set analysis via the FUMA tool identified 7 unique genes associated with traits (FDR<0.05) (Additional file 1: Supplementary Table S6). Unfortunately, further analysis of these genes failed to obtain complete biological function and MAGMA tissue-specific results.

### Drug targets in european populations: LHGS and cognitive impairment

**FIGURE 4:**
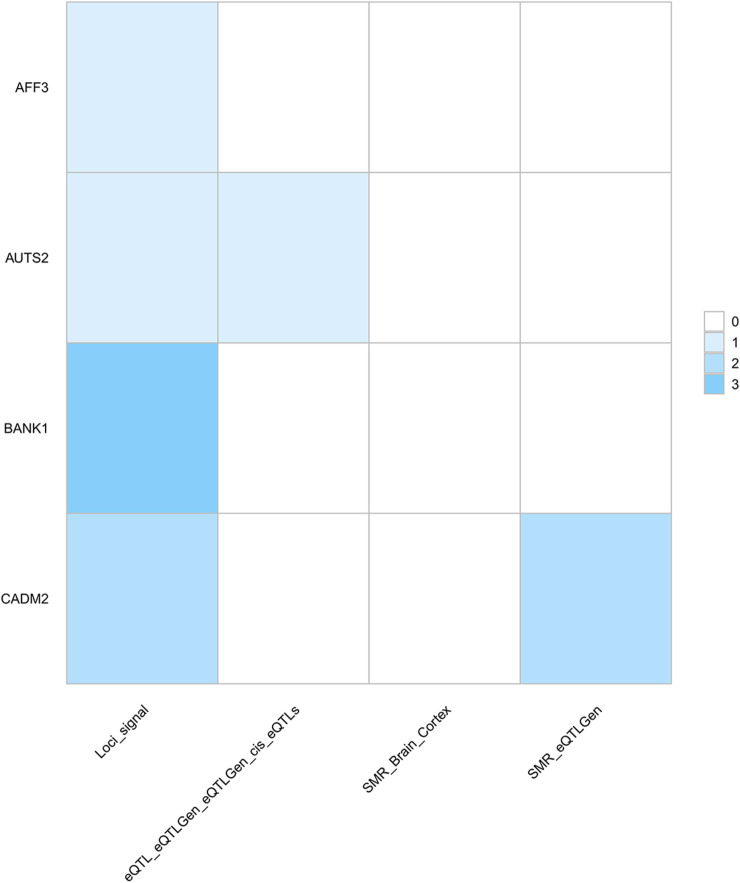
Heatmap of pleiotropic genes as potential drug targets across multiple analytical methods. Columns represent the screened genes, and rows represent different analytical methods. The color depth of each cell indicates the number of grip strength-cognitive trait pairs in which the gene is repeatedly identified by the corresponding method: darker colors indicate the gene was detected in this method across a larger number of trait pairs. The core findings of this figure are as follows: BANK1 was consistently identified across three trait pairs via PLACO; CADM2 was repeatedly detected in LHGS-CP and LHGS-INT pairs through both PLACO and SMR, highlighting their robust potential as drug targets. Notably, results from this analysis do not support an association between these targets and brain tissues. Trait abbreviations: LHGS–low hand grip strength; CP–cognitive performance; INT–intelligence; FIS–fluid intelligence score.

First, via the SMR method, we identified 3,786 potential drug targets (p_SMR < 0.05, p_HEIDI > 0.05) (Additional file 1: Supplementary Table S7). To further identify targets, we identified a set of genes significantly associated with multiple traits via PLACO analysis combined with FUMA and SMR results (Figure 4, Additional file 1: Supplementary Table S8). These genes presented significant genetic signals, and eQTL and SMR analyses further supported the pleiotropic roles of these genes in different traits and provided precise annotations for their specific locations on chromosomes.

### Immunological colocalization analyses

Some studies point to the ability of LHGS to increase cognitive impairment and identify possible mechanisms associated with chronic inflammatory responses, which suggests an important role of immune mechanisms between traits. However, we did not identify immune cells significantly associated with trait pairs by using the HyPrColoc method, which remains to be validated (Additional file 1: Supplementary Table S9).

## Conclusion

Current epidemiological studies and clinical research have confirmed the correlation between grip strength and cognitive function from different perspectives. This is also reflected in our genetic correlation analysis, on the basis of which we used a combination of methods to assess the differences in this genetic correlation at different levels and to explore the molecular mechanisms and immune mechanisms involved.

We identified a series of novel SNP loci associated with grip strength or cognitive function through PLACO. Among these novel SNP loci, rs12025777 and rs17199964 were identified in all three pairs of shapes, while rs2200465 and rs6770622 were identified in LHGS-CP-INT pairs. Previous studies have provided evidence linking certain loci to grip strength or cognitive function. For example, rs6770622 has been mentioned in a study of evolution-related genes for interspecies variation between humans and great apes and a Mendelian randomization study of cognitive and dysphagia, suggesting that it may be a key locus for human intelligence evolution and is associated with cognitive disorders (Li et al., 2020; Tian et al., 2025). It is worth noting that there is currently no direct evidence linking rs12025777, rs2200465, and rs17199964 to grip strength or cognition. To systematically assess locus novelty, we performed linkage disequilibrium (LD) expansion analysis using LDproxy (r^2^≥0.6, |D′|≥0.8 in European populations), focusing on whether our significant SNPs overlap with known loci or tag proxies in existing studies. The results show that rs4131949 (in LD with rs12025777, r^2^ = 0.778, D′ = 1.0) was identified as a candidate locus in a Parkinson’s disease multi-center study for its association with age of onset, but this association was not replicated in follow-up validation using microarray and serial analysis of gene expression (SAGE) and iterative association mapping methods (Oliveira et al., 2005); rs2200465 is in complete LD (r^2^ = 1.0, D′ = 1.0) with rs6770622 in European populations, suggesting they capture identical genetic association signals and can serve as mutual proxies; rs13105682 (in LD with rs17199964, r^2^ = 0.757, D′ = 1.0) was significantly associated (p = 2.28 × 10^−13^) with a brain region susceptible to age-related atrophy and Alzheimer’s disease pathology that influences fluid intelligence and long-term memory, based on brain structural analysis of 484 healthy participants (Manuello et al., 2024; Douaud et al., 2014). Notably, none of the LD agents of rs12025777 or rs17199964 (r^2^≥0.8) were not previously associated with grip strength and cognition in previous studies, suggesting that the loci identified may represent potential new associations.

Based on the novel SNP loci identified by PLACO, the FUMA tool further identified 7 regions and 4 genes associated with them. Among them, rs17199964 is a pleiotropic locus of BANK1 gene in 4q24 region, and bayesian colocalization analysis suggests that it is a causal locus shared between LHGS and cognitive impairment. BANK1 is associated with immune-inflammatory mechanisms, and some studies suggest it as a potential therapeutic target for immune-related diseases (Ren et al., 2021). A few studies have indicated that BANK1 influences age-related degenerative changes, but there is no direct evidence linking the gene to grip strength or cognitive function (Tanisawa et al., 2013). A GWAS study suggests that BANK1 may be associated with brain disease susceptibility (Blokland et al., 2017). In addition, there is currently a lack of direct clinical or experimental evidence for 4q24 region to relate grip strength and cognition, which has been associated with brain volume and white matter microstructural characteristics which affect cognitive performance in a GWAS post-analyses study (Ou et al., 2023).

Although the other regions were not identified by bayesian colocalization analysis, the 1p34.2 region was also thought to be shared in the three trait pairs which was associated with rs12025777 locus, and the 3p12.1 region was shared in LHGS-CP-INT which was associated with rs6770622 locus and CADM2 gene. The 1p34.2 region was shown in a clinical trial to have decreased expression of the corresponding gene in cognitive impairment diseases (Wilmot et al., 2008). Another clinical study showed that miRNA expression of 1p34.2 in the quadriceps of patients with chronic obstructive pulmonary disease was lower than that of normal subjects (Garros et al., 2017). Several GWAS analyses have linked the 3p12.1 region to differences in physical activity (de Geus, 2023). While the 3p12.1 region has been identified in a clinical proportion report as having a functional deletion that affects neurodevelopment, which can be manifested by mild intellectual disability, severe speech delay, and mild dysmorphism (Parmeggiani et al., 2018). The results of a GWAS of non-dementia older adults indicate that the CADM2 gene is associated with executive function and information processing speed, both of which are related to cognitive function (Ibrahim-Verbaas et al., 2016). At the same time, CADM2 gene expression was downregulated in an animal experiment of duchenne muscular dystrophy, which may be related to intercellular adhesion function, and its expression was also associated with other body indexes, such as body mass index and waist circumference (Granet et al., 2025; Speliotes et al., 2010).

In addition, AFF3 and AUTS2 genes are also considered to be shared genetic structures between LHGS-CP traits. Common variants of AFF3 are associated with cognition proxies such as fluid intelligence, educational attainment, and mathematical ability, whereas related studies revealed neurological deficits and grip strength loss in AFF3 knockout mouse models (Bassani et al., 2024; Voisin et al., 2021). The AUTS2 gene is named for autism susceptibility, but it affects nerve development and muscle function, and pathogenic variants can lead to symptoms such as intellectual disability, microcephaly, reduced muscle tone or limb spasms (Biel et al., 2022; Hori et al., 2021). In addition, animal experiments also showed that the expression of AUTS2 decreased in the muscle of aging rats, suggesting that this gene is related to muscle mass (Altab et al., 2025).

The SMR method was used to screen 3,786 potential drug targets, including 4 previously identified genes. At present, there is no clear record of drug data for the four targets (BANK1, CADM2, AFF3 and AUTS2) in DrugBank and Therapeutic Target Database. Only some studies suggest the possible effects of existing drugs on these targets. For example, upregulation or deletion of BANK1 was found to affect treatment outcomes in a clinical trial of irinotecan combined with rituximab for cell lymphoma, which may be related to the intervention of immune inflammatory mechanisms by drugs affecting BANK1 expression (Wang et al., 2022; Yang et al., 2018); A study on the mechanism of epithelial-mesenchymal transition and CDK4/6 inhibitor resistance in breast cancer found that CADM2 expression affects disease progression and drug efficacy, and its expression may be indirectly affected by quercetin (Li et al., 2024). In addition, it should be pointed out that because there are only computed structure models for four targets in the protein data bank, it is impossible to verify the relationship between existing drugs and targets through molecular docking. Further research is needed to promote the transformation of results in the future.

Existing studies have shown that some of the gene identified above are enriched in certain organs or tissues. For example, CADM2 is expressed throughout the central nervous system (Morris et al., 2019; Yan et al., 2018). The study aims to provide more reference value for the genetic correlation between grip strength and cognition by verifying whether the relevant loci are enriched in organs or tissues. Unfortunately, the MAGMA tissue-specific analysis failed to yield results. The S-LDSC analysis based on SNP heritability revealed inconsistent tissue enrichment for grip strength and cognition. Therefore, the current findings do not support the genetic link between grip strength and cognition at the organ or tissue level.

Similarly, previous literature has pointed out the muscle–brain crosstalk involving immune inflammatory mechanisms, the identified gene also provide evidence for this, such as BANK1 and AFF3 are risk factors for autoimmune or inflammatory diseases (Ren et al., 2021; Tsukumo et al., 2022; Gurholt et al., 2024). The study aims to verify the association between relevant loci and 731 types of immune cells, so as to provide more reference value for the immunological mechanism of grip strength and cognition. Unfortunately, no significantly related immune cells were found in the immunological colocalization analyses, so further exploration is still needed.

### Limitations

There are several limitations to our study. First, similar to many other studies, we used data at the aggregate level rather than at the individual level, which limits our ability to further stratify the population (e.g., by sex, age, or other demographic factors). Second, the limited sample size of the immune cell GWAS used in this study limits the robustness of our conclusions about the role of immune cells; therefore, caution is needed when these results are interpreted. Third, our analyses were limited to populations of European ancestry, which means that the findings may not be applicable to other populations or ancestries. In addition, the relatively small sample sizes of the main traits in our study may limit statistical efficacy, which also suggests that caution should be exercised when interpreting the results. Fourth, strict criteria limit the number of screening loci, and functional and organizational enrichment analysis around the loci fail, which further limits the explanatory power of the loci for grip strength and cognitive association. Fifth, there is currently a lack of research on the screening loci around grip strength or cognition. It is relatively novel, but lacks reliability and needs further study in the future.

### Discussion

This study revealed a complex association between grip strength and cognitive function. The results of multilevel analyses identifying pleiotropic risk loci (e.g., rs12025777, rs17199964, rs2200465 and rs6770622), pleiotropic regions (e.g., 1p34.2, 4q24 and 3p12.1) and potential drug targets (e.g., BANK1, CADM2, AFF3 and AUTS2) suggest that there may be a common mechanism between grip strength and cognitive function. These findings provide new insights into the shared genetic and biological basis for the relationship between grip strength and cognitive function.

## Data Availability

As summarized in the Methods, the data is publicly accessible and available online through the links and references provided in this study. Original contributions to the research are included in the article/supplementary materials. For further inquiries, please contact the corresponding author directly.

## Abbreviations

GWAS, genome-wide association study; SNP, single nucleotide polymorphism; PLACO, pleiotropic analysis under the composite null hypothesis; CHARLS, the China Health and Retirement Longitudinal Study; GBCS, the Guangzhou Biobank Cohort Study; DSST, the digit symbol substitution test; LDSC, linkage disequilibrium score regression; LHGS, low hand grip strength; CP, cognitive performance; CF, cognitive function; INT, intelligence; FIS, fluid intelligence score; PMR, prospective memory result; EWGSOP, the European Working Group on Sarcopenia in Older People; IVW, inverse variance weighted; MHC, major histocompatibility complex; MAF, minor allele frequency; SEs, Standard errors; S-LDSC, stratified linkage disequilibrium score regression; MSigDB, the Molecular Signatures Database; DEGs, differentially expressed genes; FUMA, functional mapping and annotation; eQTL, expression quantitative trait loci; HyPrColoc, the hypothesis prioritization for multitrait colocalization.
